# Disseminated Intravascular Coagulation Manifesting as Diffuse Alveolar Hemorrhage in a Scrub Typhus Patient: A Rarely Thought of Complication

**DOI:** 10.7759/cureus.32974

**Published:** 2022-12-26

**Authors:** Mayank Pandey, Sangita Kamath, Ajatshatru Upadhyay, Kreetee Dubey

**Affiliations:** 1 General Medicine, Tata Main Hospital, Jamshedpur, IND; 2 General Medicine, Tata Main Hospital, Jamshedpur , IND; 3 Internal Medicine, Tata Main Hospital, Jamshedpur, IND

**Keywords:** fever, alveolar hemorrhage, thrombocytopenia, scrub typhus, bleeding

## Abstract

Scrub typhus is a common cause of an acute, unexplained febrile illness. Without proper treatment, it can lead to life-threatening complications and even death. We present the case of a 16-year-old girl who presented with complaints of fever with reddish spots all over her body for 10 days and breathing difficulty for three days. She had an episode of gum bleeding just prior to admission and two episodes of hemoptysis after admission. She had severe thrombocytopenia, a low serum fibrinogen level, raised D-dimer levels, a raised activated partial thromboplastin time as well as a raised prothrombin time, and an international normalized ratio. Her chest radiograph showed diffuse bilateral interstitial infiltrates. A diagnosis of disseminated intravascular coagulation and diffuse alveolar hemorrhage secondary to possible hematological malignancy or vector-borne infectious disease was made. She was treated with intravenous doxycycline and broad-spectrum antibiotics, along with other supportive measures. Bone marrow aspiration and biopsy showed normal trilineage differentiation, normal erythropoiesis, myelopoiesis, and megakaryopoiesis. Finally, a positive immunoglobulin M (IgM) antibody for scrub typhus clinched the diagnosis. Her condition improved over the next week, and she was discharged with the advice to continue oral doxycycline for a week. This case highlights one of the rare complications of scrub typhus, disseminated intravascular coagulation, and the importance of timely initiation of treatment in such patients.

## Introduction

Scrub typhus is an infectious disease caused by Orientia tsutsugamushi, which is transmitted by the bite of the larval stage of trombiculid mites [1]. It is the most common rickettsial infection in India. In India, the disease is most prevalent in the Western and Eastern Ghats, the Himalayan foothills, which encompass the states of Maharashtra, Rajasthan, Karnataka, Kerala, Tamil Nadu, Bihar, Jharkhand, West Bengal, Meghalaya, Himachal Pradesh, and Uttaranchal. The clinical manifestations of scrub typhus may be non-specific, and it is often confused with a viral illness, malaria, or bacterial illness. It often presents with a fever, chills, headache, cough, myalgia, nausea, vomiting, and a skin rash. The disease, without proper treatment, can lead to complications such as myocarditis, hepatitis, pneumonia, acute kidney injury, meningoencephalitis, gastrointestinal bleeds, multi-organ dysfunction, and even death [2]. In our case, the patient presented with disseminated intravascular coagulation (DIC) manifesting as diffuse alveolar hemorrhage (DAH), which is a rare complication. To the best of our knowledge, this is the first case of DIC manifesting as DAH in a patient with scrub typhus in an adult reported to date. With timely intervention and proper treatment, such complications can be treated and the lives of patients can be saved.

## Case presentation

**Figure 1:**
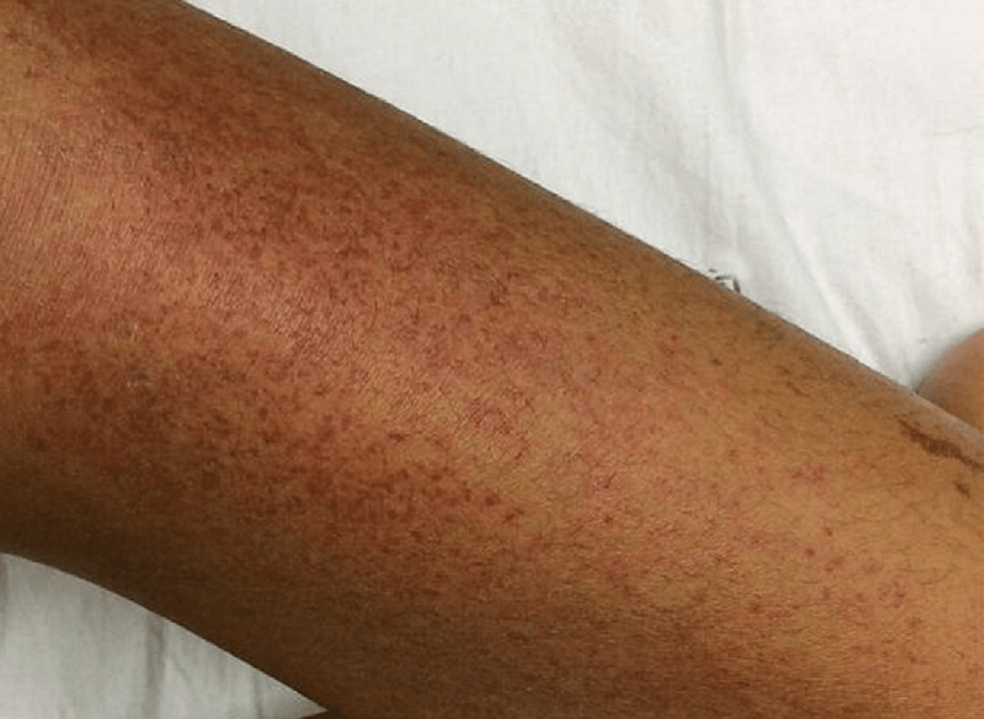
Petechial spots on the patient’s left leg.

A 16-year-old girl, a resident of Purulia in West Bengal, presented with the chief complaints of fever with reddish spots all over the body for 10 days and breathing difficulty for three days. She had an episode of gum bleeding just prior to admission. She also had two episodes of hemoptysis just after admission. On admission, she was alert, conscious, oriented, and febrile, with a temperature of 101 degrees Fahrenheit, a pulse rate of 120 beats per minute, blood pressure of 127/60 mm Hg, oxygen saturation of 85% while breathing ambient air, which improved to 98% on 10 liters of oxygen, and a respiratory rate of 30 breaths per minute. There was no lymphadenopathy, clubbing, cyanosis, or icterus. Several petechial spots were noted on the arms and legs (Figure 1), with clotted blood in the gingival cavity and gums. Respiratory system examination revealed bilateral crepitation with decreased air entry to the lung bases. The cardiovascular examination was normal except for tachycardia. The rest of the examination was normal.

**Figure 2:**
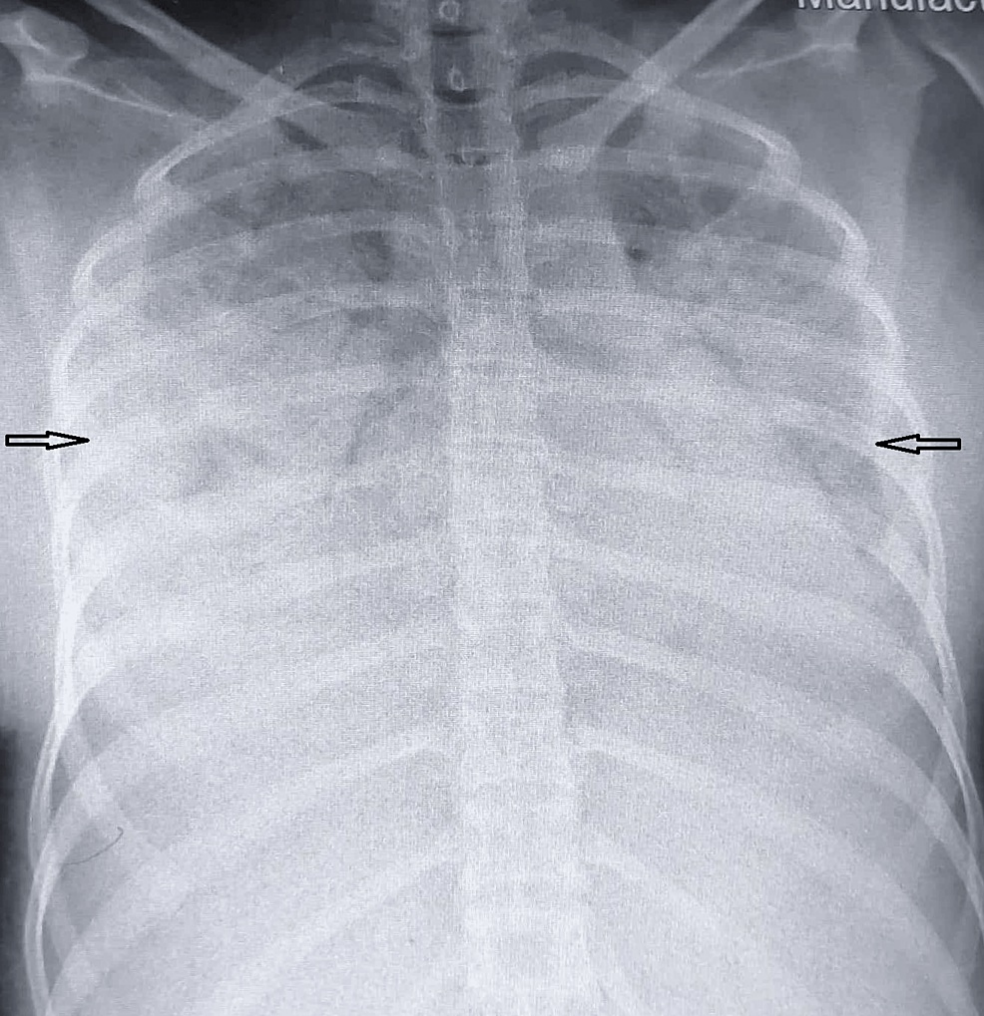
Chest radiograph on admission showing bilateral middle and lower zone opacities.

Her blood investigations showed hemoglobin of 6.5 g/dl (11.5-16.5 g/dl), a total leucocyte count of 7900/mm3 (4000-11000/mm3), and a platelet count of 14,000/mm3 (150000-450000/mm3). The serum fibrinogen level was 110 mg/dl (200-400 mg/dl), the international normalized ratio (INR) was 1.35 (0.8-1.2), the activated partial thromboplastin time was 43 seconds (25-35 seconds), the D-dimer was 2.1 micro gm/ml (less than 0.5 micro gm/ml), the C-reactive protein (CRP) was -7.06 mg/dl (0.08-0.79 mg/dl), and the liver and renal tests were normal. Tests for dengue, malaria, and chikungunya were negative. The anemia profile did not show any specific nutritional deficiencies. Direct Coomb’s test, antinuclear antibody (ANA), and anti-double stranded DNA (anti-dsDNA) were negative. Her chest radiograph showed diffuse interstitial infiltrates involving bilateral mid- and lower-zone lung fields (Figure 2). The electrocardiogram showed sinus tachycardia, and the echocardiography was normal. Ultrasonography of the abdomen showed mild ascites. A probable diagnosis of disseminated intravascular coagulation with diffuse alveolar hemorrhage secondary to possible hematological malignancy or vector-borne infectious disease was made. A bone marrow aspiration and biopsy done to rule out other causes of pancytopenia showed normal trilineage differentiation, normal erythropoiesis, myelopoiesis, and megakaryopoiesis.

She was started on broad-spectrum antibiotics along with doxycycline to cover for rickettsial infections, along with other supportive measures, including platelet transfusion (one unit of single donor platelets) and packed red blood cell transfusion. Suspecting vector-borne infectious disease, immunoglobulin M (IgM) antibodies against O. tsutsugamushi and Leptospira by ELISA were advised. 

**Figure 3:**
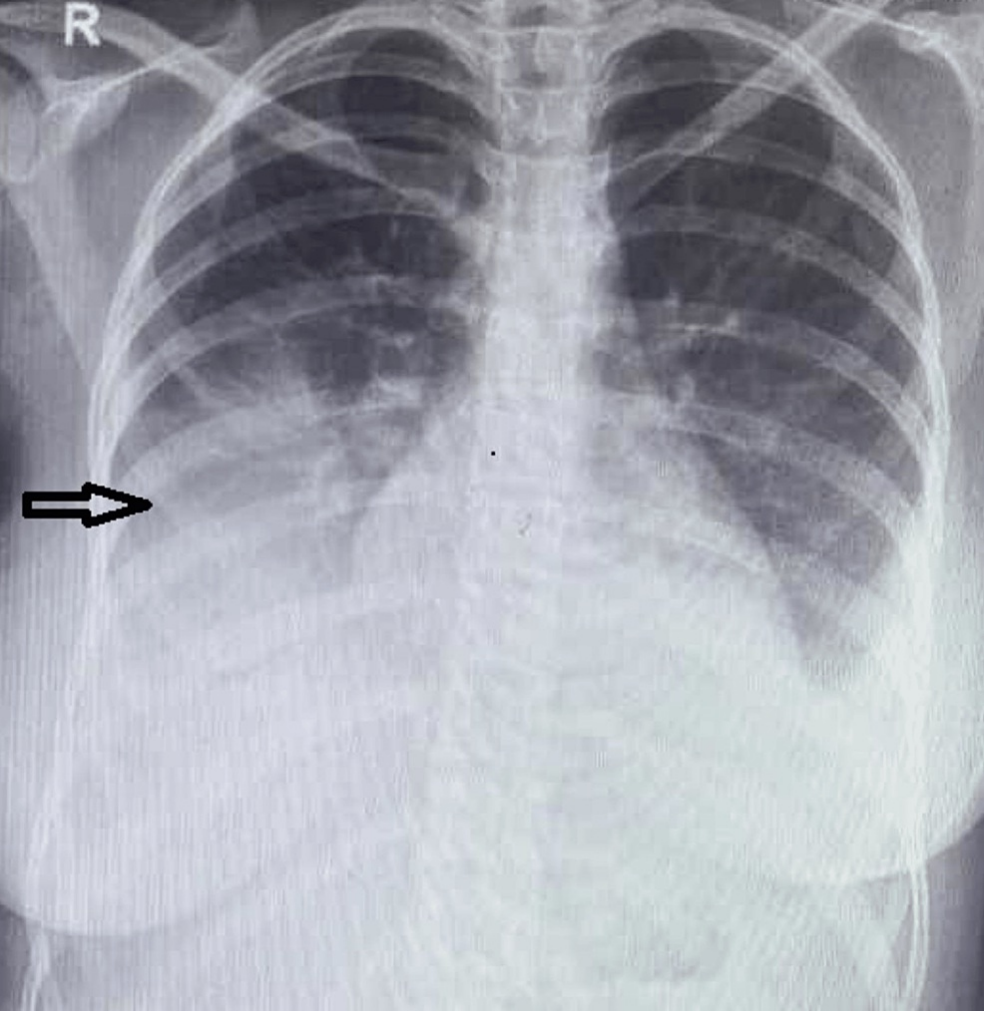
Repeat chest radiograph after three days, showing significant improvement in the opacities.

Her condition improved over the next three days. Her repeat chest radiograph (Figure 3) showed marked improvement; she was off oxygen support.

**Figure 4:**
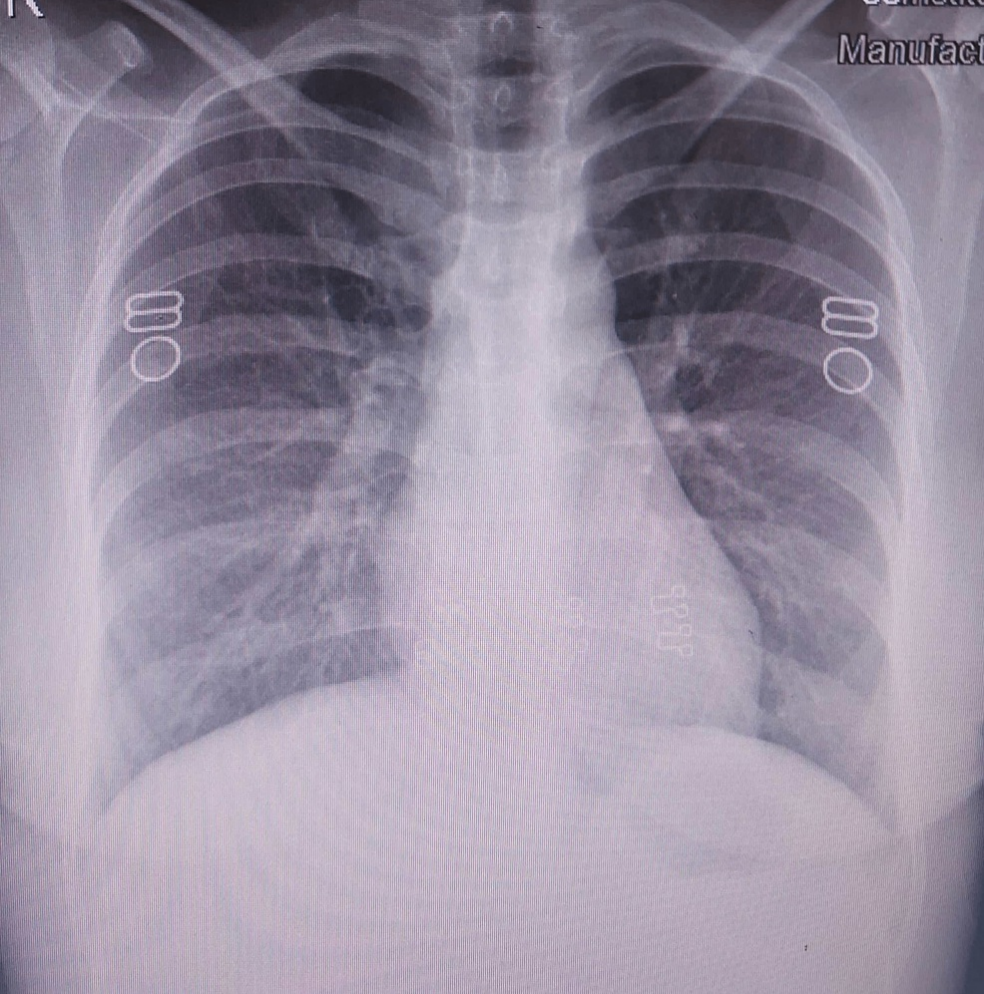
Repeat chest radiograph at the time of discharge showing clear lung fields.

Her platelet count continued to improve and increased to 71,000/mm3. Her IgM antibody levels came out to be positive for scrub typhus. She was discharged after completing seven days of intravenous doxycycline with the advice to continue oral doxycycline for a further seven days. Her chest radiograph at the time of discharge (Figure 4) showed clear lung fields.

## Discussion

Scrub typhus is a vector-borne zoonotic infection and one of the most common rickettsial infections worldwide. The disease is endemic in Southeast Asia, East Asia, the Pacific Islands, and Northern Australia (the Tsutsugamushi Triangle) [3]. However, cases have also been reported from Chile, Peru, Africa, and the Arabian Peninsula. In India, after the early epidemic during the Second World War in the states of Assam and Bengal, there has been a re-emergence of cases in recent years [4].

Man is accidentally infected when he encroaches on the mite-infested areas, known as the mite islands. These areas consist of areas with secondary scrub growth, which grows after the clearance of primary forest, hence the term "scrub typhus." However, the infection can occur in diverse habitats such as the seashore, rice fields, and even semi-deserts [1,5].

Fever is the most common feature of scrub typhus, and in endemic areas, it is one of the causes of "fever of unknown origin." The clinical manifestations of this disease range from subclinical disease to organ failure to fatal disease [1]. After ruling out complicated malaria, leptospirosis, and dengue fever, many of these cases remain undiagnosed [6].

Eschar is the typical skin rash found in scrub typhus. Though it may be found in other conditions, its presence in the setting of unexplained febrile illness is pathognomonic of scrub typhus. It begins as a painless papule, which evolves into a dark-colored scab-like scar. It is often found in the groin, axilla, genitalia, and neck. The frequency of eschar varies from 7 to 80% in patients infected with scrub typhus, but its occurrence is rare in the southeast Asian population [2]. We did not find any eschar in our case.

Serious complications of scrub typhus are not uncommon and may be fatal; they include pneumonia, myocarditis, meningoencephalitis, acute renal failure, and gastrointestinal bleeding. Early diagnosis is important because there is usually an excellent response to treatment, and timely antimicrobial therapy may help prevent complications. In developing countries with limited diagnostic facilities, it is prudent to recommend empiric therapy in patients with undifferentiated febrile illnesses who have evidence of multiple system involvement [7].

Antibody titers equal to or greater than 1:360 on one occasion for the OX-K antigen or a four-fold increase on two different occasions are considered diagnostic [4,5]. However, as this test has poor sensitivity and specificity, it is fast becoming obsolete. Serologic tests like indirect immunofluorescence or enzyme-linked immunosorbent assay (ELISA) to detect IgM O tsutsugamushi antibodies are used nowadays, with molecular tests such as quantitative polymerase chain reaction (PCR) being restricted to high resource settings [2].

Doxycycline 200 mg/day is the treatment of choice for scrub typhus. Other antibiotics useful for the treatment of this infection are chloramphenicol, azithromycin, and rifampicin. Rapid resolution of fever following doxycycline is so characteristic that it can be used as a therapeutic test [1,5].

Our case was unique in the sense that the patient presented with bleeding manifestations due to severe thrombocytopenia secondary to DIC, which is usually not thought of in scrub typhus. Yuko et al. have described a case of scrub typhus complicated by severe disseminated intravascular coagulation (DIC), where the patient succumbed and DIC was later confirmed by autopsy [8]. In a retrospective study by Lee HJ et al., they found that 18 out of 365 patients had bleeding manifestations, of which 13 had DIC. They concluded that activation of the coagulation system is an important feature of scrub typhus and correlates with severe disease, including bleeding [9]. Yun-Ho Lin et al. have described a case of DIC complicated by hemophagocytic syndrome. The patient was found to have scrub typhus, as confirmed by a positive Weil-Felix test, the presence of IgM and IgG antibodies, and a positive PCR for O. tsutsugamushi, but could not be saved despite treatment [10]. Izumo T et al. described a case of scrub typhus with DIC and acute respiratory distress syndrome (ARDS), and the patient’s condition significantly improved after the administration of tetracycline [11]. In a study of the profile of scrub typhus in a tertiary care hospital by Kamath SD et al., only one out of 42 patients developed DIC [12].

We started treatment with doxycycline from the very first day, awaiting an IgM antibody report for scrub typhus, on a high index of suspicion because the patient came from an endemic region (Purulia, West Bengal). The patient’s unusual presentation with negative tests for dengue, malaria, a lack of eschar, and a negative ANA made it imperative to exclude a hematological malignancy as a cause for the DIC. Bone marrow aspiration and biopsy did not show evidence of malignancy or immune thrombocytopenic purpura (ITP). Finally, a positive IgM antibody for O. tsutsugamushi clinched the diagnosis.

## Conclusions

Disseminated intravascular coagulation remains a rare complication of scrub typhus. The typical eschar that is pathognomonic for scrub typhus may not always be present in all patients. It is prudent to consider a diagnosis of scrub typhus in patients who have negative tests for dengue, malaria, and typhoid. Early treatment with doxycycline is important to prevent fatal complications. Rapid resolution of fever following doxycycline therapy can be used as a surrogate test for scrub typhus. All clinicians should know about the rare complications of this reemerging disease in order to initiate timely therapy and thus prevent fatal outcomes.
